# Supplementary material for: Integrating mental health into primary care in Nigeria: Implementation outcomes and clinical impact of the HAPPINESS intervention
Source: Glob Ment Health (Camb). 2024 Jan 12;11:e9. doi: 10.1017/gmh.2024.4 (PMC10882178; doi:10.1017/gmh.2024.4)
Supplement: Iheanacho et al. supplementary material 1 — Iheanacho et al. supplementary material [file S2054425124000049sup001.pdf]

The HAPPINESS Project

Health Action for Psychiatric Problems in Nigeria including Epilepsy and SubstanceS

Clinical toolkit for screening, monitoring, and record keeping

Table of Contents

Individual Client Records

Enrollment form 2

Routine follow up 7

Periodic review 9

Monitoring Tools

PHQ for depression 13

BPRS for psychosis 14

Clinic Recordkeeping

Standard operating procedure 15

Monthly reporting form 29

Enrolment Form

Client ID ☐☐☐☐☐☐☐

COMPLETE THIS FORM TO ENROL THE CLIENT IN THE PROGRAMME

A. ADMINISTRATIVE INFORMATION

1. **Client ID** ☐☐☐☐☐☐☐

2. **New** ☐ New ☐ Existing

3. **Date of registration with Programme**
D D / M M / Y Y Y Y

4. **Has client given consent for data to be stored on the database?**
☐ Yes ☐ No

5a. **First name(s)**

5b. **Surname**

6a. **Region**

6b. **Division**

6c. **Community**

7a. **Contact name**

7b. **Contact telephone**

7c. **Contact relationship to client**

8a. **Nurse/clinician**

8b. **Volunteer/aide** (write "none" if not applicable)

B. DEMOGRAPHIC INFORMATION

9a. **Date of birth** (or nearest approximation)
D D M M Y Y Y Y

9b. **Age** (specify months for infants <1 year only)
0 0 0 ☐ Years ☐ Months

10. **Gender** ☐ Male ☐ Female

11. **Number of children** 0 0

12. **Education level** (choose one)
- ☐ None/not completed primary
- ☐ Literacy classes/literate
- ☐ Completed primary school
- ☐ Completed secondary school
- ☐ Further education

13. **Marital status** (choose one)
- ☐ Never married
- ☐ Co-habiting
- ☐ Married
- ☐ Separated
- ☐ Divorced
- ☐ Widowed

14a. **Current occupation** (choose one)
- ☐ Pre-school/nursery
- ☐ Student/training
- ☐ Unemployed
- ☐ Keeping house/homemaker
- ☐ Farming/petty trading
- ☐ Manual worker
- ☐ Non-manual worker
- ☐ Professional
- ☐ Retired

14b. **Specify exact current occupation**

Enrolment Form

Client ID

C. INFORMATION ABOUT THE PROBLEM

15. **Problem category** (tick all the problem categories that apply to this client)

Mental/Neurological	*Physical*	*Other/None*
X Mental illness (psychosocial impairment)	X Low vision/blindness	X No impairment
X Intellectual impairment	X Hearing impairment/deafness	X Other impairment (specify below)
X Epilepsy	X Other physical impairment (specify below)	

If "other physical" or "other impairment" is ticked, please specify:

16a. **Main problem** (circle most important problem to client in above list)

16b. **Main Problem duration** (specify months for duration <1 year only)

0 0 X Years X Months

D. DIAGNOSIS AND SEVERITY OF CONDITION

17. **Medical history.** Please make notes on the history of the patient's problem, for example the background to the problem, for epilepsy, how often the seizures occur and the type of epilepsy. Complete on additional sheets if necessary and attach.

Enrolment Form

Client ID

18. **Physical and mental state examination.** Complete on additional sheets if necessary and attach.

19a. **Mental health diagnosis.** Put a number in all the boxes that in your clinical judgment, the patient is suffering from, starting with 1 for the most important problem, 2 for the second most important etc. Please also specify the exact diagnosis in box 20b. for example "drug induced psychosis" or "schizophrenia". For alcohol/substance abuse, please state which substance in box 20b.

Adolescents and adults (primarily)	*Children and elderly (primarily)*	*Other/All age groups*
0 Depression/Anxiety	0 Child mental disorder	0 Epilepsy
0 Alcohol/Substance abuse	0 Dementia	0 Other (specify in box below)
0 Psychosis (including schizophrenia, manic depression/bipolar)		

19b. **Specify exact diagnosis/substance used**

If depression is suspected and patient is age 18 or over, complete additional PHQ-9. If patient is diagnosed with schizophrenia, complete additional CGI-Sch.

20. **Clinical Global Impression Severity (CGI-S).** Considering your total clinical experience with this particular population, how mentally ill is the patient at this time? Complete for all patients.

Normal, not at all ill	*Borderline mentally ill*	*Mildly ill*	*Moderately ill*	*Markedly ill*	*Severely ill*	*Among the most extremely ill*
1	2	3	4	5	6	7

21. **Frequency of seizures.** Write 00 if no seizures in past month or patient does not have epilepsy.

0 0 seizures in past month

E. FUNCTIONING AND QUALITY OF LIFE

22. **Global Assessment of Function Scale.** Enter a score between 0 and 100 based on your clinical judgement of the patient. *Refer to the GAF SCORING SHEET for CHILDREN OR ADULTS as appropriate.*

0 0 0 GAF score (0-100)

23. **Quality of Life.** Record any comments you have on the patient's quality of life.

F. FAMILY ATTITUDES TOWARDS PROBLEM

24. **Attitude of family toward problem** (tick one)

- X Positive (trying to change attitudes of others)
- X Accepting (open about problem)
- X Ashamed (trying to hide problem from others)

25. **Willingness of family to help patient** (tick one)

- X Willing to help
- X Reluctant to help
- X Refusing to help

G. REHABILITATION PLAN

26. **Medication prescribed.** Use generic names. Tick all that apply.

Anti-psychotics	*Anti-epileptics*	*Anti-depressants*	*Other/None*
X Chlorpromazine	X Carbamazepine	X Amitriptyline	X Diazepam
X Haloperidol	X Phenobarbital	X Fluoxetine	X Benzhexol
X Fluphenazine Decanoate			X None (skip to 32)
X Trifluoperazine			X Other (specify in box below)
X Risperidone			

If "other" is ticked, please specify

27. **Prescription details**

	Name of medication	Route	Dose	Frequency per day	Number of days
Medication 1		X Tablets (Oral)<br>X Injection (Parenteral)			
Medication 2		X Tablets (Oral)<br>X Injection (Parenteral)			
Medication 3		X Tablets (Oral)<br>X Injection (Parenteral)			

28. **Total cost of medication**

Medication 1		Medication 2		Medication 3		Total
0	+	0	+	0	=	0

29. **Total paid for medication** (if no payment, record as 0)

0 NGN

30. **Interventions administered by clinician or aide.** Tick all that apply or "none."

- X None
- X Family education/support
- X Client counselling (e.g. problem-solving therapy)
- X Parenting skills training
- X Daily living skills training
- X Other (specify)

If "other" is ticked, please specify:

31. **Referred for other services.** Tick all that apply or "none."

- X None
- X Self-help/peer support groups
- X Educational support/return to school
- X Legal support/police
- X External mental health services
- X Other medical care (specify)
- X Other non-medical care (specify)

If "other medical" or "other non-medical" is ticked, please specify:

H. NOTES/ANY OTHER RELEVANT INFORMATION *(Please continue on a separate sheet if necessary)*

Routine Follow-up Form

Patient ID | | | | | | | |

USE THIS FORM FOR ANY ROUTINE FOLLOW UP VISITS EXCEPT 6 MONTH FOLLOW UP WHEN SPECIFIC 'PERIODIC REVIEW FORM' SHOULD BE USED.

First name

Family Name

Date today

Date of enrolment interview

VISIT 1:

Progress since last review: *Social, physical* and *Psychological*

Current medication: *Is patient willing to take medication? Any side effects?*

Any other issues:

Plan until next review:

Signed:
(Name and position)

VISIT 2:

Progress since last review: *Social, physical* and *Psychological*

Current medication: *Is patient willing to take medication? Any side effects?*

Any other issues:

Plan until next review:

Signed:
(Name and position)

Routine Follow-up Form Patient ID

VISIT 3:

Progress since last review: *Social, physical* and *Psychological*

Current medication: *Is patient willing to take medication? Any side effects?*

Any other issues:

Plan until next review:

Signed:
(Name and position)

VISIT 4:

Progress since last review: *Social, physical* and *Psychological*

Current medication: *Is patient willing to take medication? Any side effects?*

Any other issues:

Plan until next review:

Signed:
(Name and position)

Periodic Review Form

Client ID ☐☐☐☐☐☐☐

COMPLETE THIS FORM EVERY SIX MONTHS AFTER A PATIENT IS ENROLLED IN THE PROGRAMME

A. ADMINISTRATIVE INFORMATION

1. **Client ID** ☐☐☐☐☐☐☐

2. **Date of this review** D D / M M / Y Y Y Y

3a. **Nurse/clinician**

3b. **Volunteer/aide**

4a. **New contact name** (if changed)

4b. **New contact telephone** (if changed)

If contact information does not need to be updated, skip to 5a.

B. REHABILITATION PLAN OVER PAST 6 MONTHS

5a. **Has there been a change in the type of medication prescribed in the past 6 months?** Tick one.

- X Yes
- X No

If "No" is ticked, skip to 6.

5b. **If yes, why was the change made?** Tick all reasons that apply.

- X Side effects
- X Cost
- X Patient choice
- X Ineffectiveness
- X Other (Specify reason below)

6. **Over the past 6 months, how often has the patient/family been able to pay for their medication?** Tick one.

- X All of the time
- X More than half the time
- X Less than half the time
- X None of the time

7a. **In the past month, how often has the patient been taking the medicine as prescribed?** Tick one.

- X All of the time (every day, full prescribed dosage)
- X More than half the time
- X Less than half the time
- X None of the time

If "All of the time" is ticked, skip to 8a.

7b. **If patient did not take the medicine as prescribed all of the time, why not?** Tick all that apply

- X Side effects
- X Cost
- X Patient choice
- X Ineffectiveness
- X Missed appointment
- X Medication not available
- X Other (Specify reason below)

8a. **How severe are the side effects experienced by the patient of the prescribed medicine?** Tick one.

- X No side effects
- X Mild (does not significantly interfere with functioning)
- X Moderate (interferes with functioning)
- X Severe (side effects outweigh therapeutic effect)

If "No side effects" is ticked, skip to 9.

Periodic Review Form

Client ID | | | | | | | |

8b. **Comment on any side effects the patient is experiencing.** Complete on additional sheets if necessary and attach.

9. **Which psychosocial interventions were received in the last 6 months?** Tick all that apply or "None".

- X None
- X Family education/support
- X Client counselling (e.g. problem-solving therapy)
- X Other (specify intervention below)

10. **Which referral services were accessed in the last 6 months?** Tick all that apply or "None".

- X None
- X Self-help/peer support groups
- X Educational support/return to school
- X Legal support/police
- X External mental health services
- X Other medical care (specify)
- X Other non-medical care (specify)

If "other medical" or "other non-medical" is ticked, please specify:

C. CLINICAL OUTCOMES

11. **Clinical Global Impression Severity (CGI-S).** Considering your total clinical experience with this particular population, how mentally ill is the patient at this time? Complete for all patients.

Normal, not at all ill	*Borderline mentally ill*	*Mildly ill*	*Moderately ill*	*Markedly ill*	*Severely ill*	*Among the most extremely ill*
1	2	3	4	5	6	7

12. **Frequency of seizures.** Write 00 if no seizures in past month or patient does not have epilepsy.

| 0 | 0 |

***If depression is suspected and patient is age 18 or over, complete additional PHQ-9. If patient is diagnosed with schizophrenia, complete additional CGI-Sch.**

D. FUNCTIONING AND QUALITY OF LIFE

13. **Global Assessment of Function Scale.** Enter a score between 0 and 100 based on your clinical judgement of the patient. *Refer to the GAF SCORING SHEET for CHILDREN OR ADULTS as appropriate.*

| 0 | 0 | GAF score (0-100)

Periodic Review Form

Client ID | | | | | | | |

14. **Quality of Life.** Record any comments you have on the patient's quality of life. Complete on additional sheets if necessary and attach.

E. SOCIAL OUTCOMES

15. **Attitude of family toward problem** (tick one)

- X Positive (trying to change attitudes of others)
- X Accepting (open about problem)
- X Ashamed (trying to hide problem from others)

16. **Willingness of family to help patient** (tick one)

- X Willing to help
- X Reluctant to help
- X Refusing to help

17. **How have the following things changed in the patients' life in the past 6 months?** Comment on any change in box provided.

	Changed for better	*No change*	*Change for worse*	Comments
Interpersonal relationships	X	X	X	
Education/training	X	X	X	
Job/occupation	X	X	X	
Treatment by others/discrimination	X	X	X	
Other (Specify what changed below)	X	X	X	

F. REHABILITATION PLAN FOR NEXT 6 MONTHS

18. **Are you changing the rehabilitation plan as a result of this 6 month review?** X Yes X No

19. **Medication prescribed.** Use generic names. Tick all that apply.

Anti-psychotics	*Anti-epileptics*	*Anti-depressants*	*Other/None*
X Chlorpromazine	X Carbamazepine	X Amitriptyline	X Diazepam
X Haloperidol	X Phenobarbital	X Fluoxetine	X Benzhexol
X Fluphenazine Decanoate			X None (skip to 32)
X Trifluoperazine			X Other (specify below)
X Risperidone			

20. **Prescription details**

	Name of medication	Route	Dose	Frequency per day	Number of days
Medication 1		X Tablets (Oral)<br>X Injection (Parenteral)			
Medication 2		X Tablets (Oral)<br>X Injection (Parenteral)			

	Name of medication	Route	Dose	Frequency per day	Number of days
Medication 3		X Tablets (Oral)<br>X Injection (Parenteral)			

21. **Total cost of medications**

Medication 1		Medication 2		Medication 3		Total
0	+	0	+	0	=	0

22. **Total paid for medications** (if no payment, record as 0)

0 NGN

23. **Interventions administered by clinician or aide.** Tick all that apply or "none."

- X None
- X Family education/support
- X Client counselling (e.g. problem-solving therapy)
- X Other (specify intervention below)

24. **Referred for other services.** Tick all that apply or "none."

- X None
- X Self-help/peer support groups
- X Educational support/return to school
- X Legal support/police
- X External mental health services
- X Other medical care (specify)
- X Other non-medical care (specify)

If "other medical" or "other non-medical" is ticked, please specify:

G. NOTES/ANY OTHER RELEVANT INFORMATION *(Please continue on a separate sheet if necessary)*

PHQ-9: Symptoms of depression* *(complete for all clients)*

Over the *last 2 weeks,* how often have you been bothered by any of the following problems? *(use "✓" to indicate your answer)*	Not at all	Several days	More than half the days	Nearly every day
1. Little interest or pleasure in doing things	0	1	2	3
2. Feeling down, depressed, or hopeless	0	1	2	3
3. Trouble falling or staying asleep, or sleeping too much	0	1	2	3
4. Feeling tired or having little energy	0	1	2	3
5. Poor appetite or overeating	0	1	2	3
6. Feeling bad about yourself—or that you are a failure or have let yourself or your family down	0	1	2	3
7. Trouble concentrating on things, such as reading the newspaper or watching television	0	1	2	3
8. Moving or speaking so slowly that other people could have noticed. Or the opposite—being so fidgety or restless that you have been moving around a lot more than usual	0	1	2	3
9. Thoughts that you would be better off dead, or of hurting yourself in some way	0	1	2	3
Add columns:		+	+	
(For interpretation of TOTAL, please refer to accompanying scoring card) Total:				

Total Score	Depression Severity
1-4	Minimal depression
5-9	Mild depression
10-14	Moderate depression
15-19	Moderately severe depression
20-27	Severe depression

Brief Psychiatric Rating Scale (BPRS)

Patient Name ______________________________ Today's Date ______________

Please enter the score for the term that best describes the patient's condition.

0 = Not assessed, 1 = Not present, 2 = Very mild, 3 = Mild, 4 = Moderate, 5 = Moderately severe, 6 = Severe, 7 = Extremely severe

Score		
☐	1.	SOMATIC CONCERN<br>Preoccupation with physical health, fear of physical illness, hypochondriasis.
☐	2.	ANXIETY<br>Worry, fear, over-concern for present or future, uneasiness.
☐	3.	EMOTIONAL WITHDRAWAL<br>Lack of spontaneous interaction, isolation deficiency in relating to others.
☐	4.	CONCEPTUAL DISORGANIZATION<br>Thought processes confused, disconnected, disorganized, disrupted.
☐	5.	GUILT FEELINGS<br>Self-blame, shame, remorse for past behavior.
☐	6.	TENSION<br>Physical and motor manifestations of nervousness, over-activation.
☐	7.	MANNERISMS AND POSTURING<br>Peculiar, bizarre, unnatural motor behavior (not including tic).
☐	8.	GRANDIOSITY<br>Exaggerated self-opinion, arrogance, conviction of unusual power or abilities.
☐	9.	DEPRESSIVE MOOD<br>Sorrow, sadness, despondency, pessimism.
☐	10.	HOSTILITY<br>Animosity, contempt, belligerence, disdain for others.
☐	11.	SUSPICIOUSNESS<br>Mistrust, belief others harbor malicious or discriminatory intent.
☐	12.	HALLUCINATORY BEHAVIOR<br>Perceptions without normal external stimulus correspondence.
☐	13.	MOTOR RETARDATION<br>Slowed, weakened movements or speech, reduced body tone.
☐	14.	UNCOOPERATIVENESS<br>Resistance, guardedness, rejection of authority.
☐	15.	UNUSUAL THOUGHT CONTENT<br>Unusual, odd, strange, bizarre thought content.
☐	16.	BLUNTED AFFECT<br>Reduced emotional tone, reduction in formal intensity of feelings, flatness.
☐	17.	EXCITEMENT<br>Heightened emotional tone, agitation, increased reactivity.
☐	18.	DISORIENTATION<br>Confusion or lack of proper association for person, place or time.

Standard Operating Procedures:

Clinical Record-Keeping and Reporting for Community Psychiatric Nurses

I. Getting started

Effective record-keeping and reporting are crucial, not only to ensure accountability, transparency, and good clinical decision-making, but also to demonstrate the value of your services for the purposes of wider research, evaluation, and advocacy. However, community-based mental health services are relatively new in Nigeria, and most Community Psychiatric Nurses (CPNs) won't have experience setting up an office record-keeping and reporting system that can accommodate the many unique needs of mental health service users, providers, donors, and other stakeholders.

These standard operating procedures (SOPs) are designed as a reference to help you get started. They cannot substitute for adequate training and supervision, but they may help you to better understand what is expected of you in your administrative role as a CPN.

In the next section, "**What you need**", you will find a directory of the records and tools you will need to have available, as well as the equipment you will need to access in order to use and to store them.

In section III, "**Getting organized**," you will be given advice on how to organize and store your files.

Section IV, "**Recording your services**," will guide you through the record-keeping steps you will go through as you serve your clients.

Section V, "**Reporting your services**," further explains what you will do to compile your first monthly report of your services.

Each section ends with a Q&A section, for common issues you may encounter as you begin record-keeping at your new clinic.

II. What you need

A. Directory of records:

Every CPN should be equipped with the following records in order to facilitate effective record-keeping:

1. **Appointment cards** (Appendix 1): Appointment cards should be relatively small and portable, and printed on durable paper, so that patients can take them home for reference. At a minimum, they should include the patient name, ID no., and a space to write appointment dates. If general appointment cards are not already available for use by your clinic, you can print your own using the given format.
2. **Daily register** (Appendix 2): In the absence of a formal Nigerian Health Information System (HIS) register, this may be a series of print-outs bound together, or a notebook carefully set up to follow the given format. This register is divided into two parts:
    a. **New client register**
    b. **Existing client register**
3. **Monthly reporting forms** (Appendix 3): In the absence of a formal Nigerian Health Information System (HIS) reporting form, the given format may be printed and used.
4. **Patient files with logs** (Appendix 4): These should be durable office files, clearly printed with the clinic name and patient ID no. Ideally, a file log will also be printed on the file cover. In the absence of printed files, the given format may be printed and affixed to the *interior* of the file, with the client ID no. clearly written on the *exterior* of the file.
5. **Enrolment forms:** These are multi-page formats which may be printed front-to-back to save paper. Each multi-page form should be stapled.
6. **Follow-up forms:** These are single page formats which may be printed front-to-back to save paper (as multiple follow-up forms will generally accumulate over the course of a patient's treatment).
7. **6-month review forms**: These are multi-page formats which may be printed front-to-back to save paper. Each multi-page form should be stapled.
8. **Referral register**: In the absence of a formal register, this again may be a series of print-outs bound together, or a notebook carefully set up to follow the given format.
9. **Referral forms**: These are single-page formats, including a half-page referral section (top) and half-page back-referral section (bottom). They should *not* be printed front-to-back.

B. Equipment checklist:

The CPN should have access to the following equipment in order to facilitate effective record-keeping using the given records, tools, and procedures:

1. **Secure office space:** Confidential patient files should be locked in a secure office space when not in use.
2. **Stapler and scissors**: These are necessary to collate patient forms, and to separate the two sections of the referral forms.
3. **Pens (not pencils)**: These must be capable of providing a clear impression on carbon copies of receipts.
4. **Additional files/envelopes:** These should be used to store additional tools and records that should not otherwise be stored in the patient files.

III. Getting organized

A. Storing clinical records

Do your best to keep all the necessary clinical forms for one patient in one file (see section IV, "Recording your services"). Do not mix forms for multiple patients in a single file. The log on your patient files (see section IV) will help you to double-check that no forms are missing from a folder.

Do not throw away clinical records, even if the patient has been lost to follow-up or referred to another clinic to continue services. As much as possible, try to keep your files away from dirt, moisture, and direct sunlight.

Common Questions

Q1: *Why should I organize my files by ID number?*

A1: Each patient is assigned a unique ID number—one number for each patient, given in sequential order. That number is used to track the patient's information on everything from clinical records to receipts to database entries for research. Two people may have the same name, or one person may spell a name in multiple ways, so this ID is a more reliable and secure way to link information contained in different sources. When a query is raised about a patient's information on a record (for example, on a receipt for a prescription) you may want to check the more detailed information collected in the patient's file (such as the diagnosis of the patient receiving the prescription). This is why all patient files should be consistently organized in order of patient ID number.

Q2: *What if I somehow end up with more than one file for a patient?*

A2: If you end up with two files on the same patient (e.g., if you accumulate too many records to fit in a single file), clearly label on the front of the first file "file 1 of 2", and on the second file "file 2 of 2". Be certain to include the patient ID on each of the two files.

Q3: *What if someone requests a patient's file?*

A3: It is the program's responsibility to maintain accurate records on all service users and to ensure the confidentiality of patient information.

IV. Recording your services

A. Enrolling a new client

1. When a new client arrives at your services, you should first open a new patient file by filling out a **patient file log** (Appendix) with the following:
    a. Patient ID no.
    b. Clinic name*
    c. Visit date* (today's date, recorded in row 1, the shaded row with "Enrollment" as the "Visit Type")
2. If this file log is not already printed on the cover of the patient file, staple it to the inside cover of the file, and write the patient ID no. clearly on the outside of the file.*
3. Complete an **enrollment form**, being certain to answer every question and label each page with the patient ID no., as instructed in the **guide to patient forms**. You might also want to refer to the **GAF scoring tool** to help you complete this section of the tool. When complete, place the enrollment form inside the patient file.
4. Issue an **appointment card** to the new client, by completing the following:
    a. Clinic name*
    b. Patient name*
    c. Patient ID no.*
    d. Any crucial notes (e.g. clinic phone number in case of emergency)
    e. Today's date* (next to "1" on the reverse side of the appointment card)
    f. Next appointment date (next to "2" on the reverse side of the appointment card)
5. Copy the required information from the enrollment form to the **new client register**, in your **daily register.**

B. Following up on an existing client

1. For any existing client who arrives at your services, you should take the client's **appointment card** and use the patient ID no. recorded on the card to locate the patient's file.*
2. Examine the **patient file log**. If it has been less than six months since the client's last 6-month review or enrollment visit, update the log with the following:
    a. Visit date (today's date)
    b. Visit type (tick "follow-up")
3. Complete a **follow-up form**, being certain to answer every question and label each page with the patient ID no. Be sure to note any changes to the prescription, including dosage and cost, since the last visit. When complete, place the follow-up form inside the patient file.
4. Update the **appointment card** for the client, by adding the next scheduled appointment date in the next available row on the reverse side of the card.
5. Add the required information to the **existing client register** of the **daily register**, using information from the patient's enrollment form and the follow-up form you just completed. For "Visit Type," look back to the patient file log to see whether this is the patient's first follow-up visit this month. If it is, tick the cell for "First follow-up of the month". If it isn't, tick the other cell for "Extra follow-up this month".

C. 6-month review of on an existing client

1. For any existing client who arrives at your services, you should take the client's **appointment card** and use the patient ID no. recorded on the card to locate the patient's file.
2. Examine the **patient file log**. If it has been six months or more since the client's last 6-month review or enrollment visit, update the log with the following:
    c. Visit date (today's date)

   d. Visit type (tick "6-Month Review")
3. Complete a **6-month review form**, being certain to answer every question and label each page with the patient ID no. You might also want to refer to the **GAF scoring tool** (Appendix) to help you complete this section of the tool. When complete, place the 6-month review form inside the patient file.
4. Update the **appointment card** for the client, by adding the next scheduled appointment date in the next available row on the reverse side of the card.
5. Add the required information to the **daily register** using information from the patient's enrollment form and the 6-month review form you just completed. For "Visit Type," look back to the patient file log to see whether this is the patient's first visit this month. If it is, tick the cell for "First follow-up of the month". If it isn't, tick the other cell for "Extra follow-up this month".

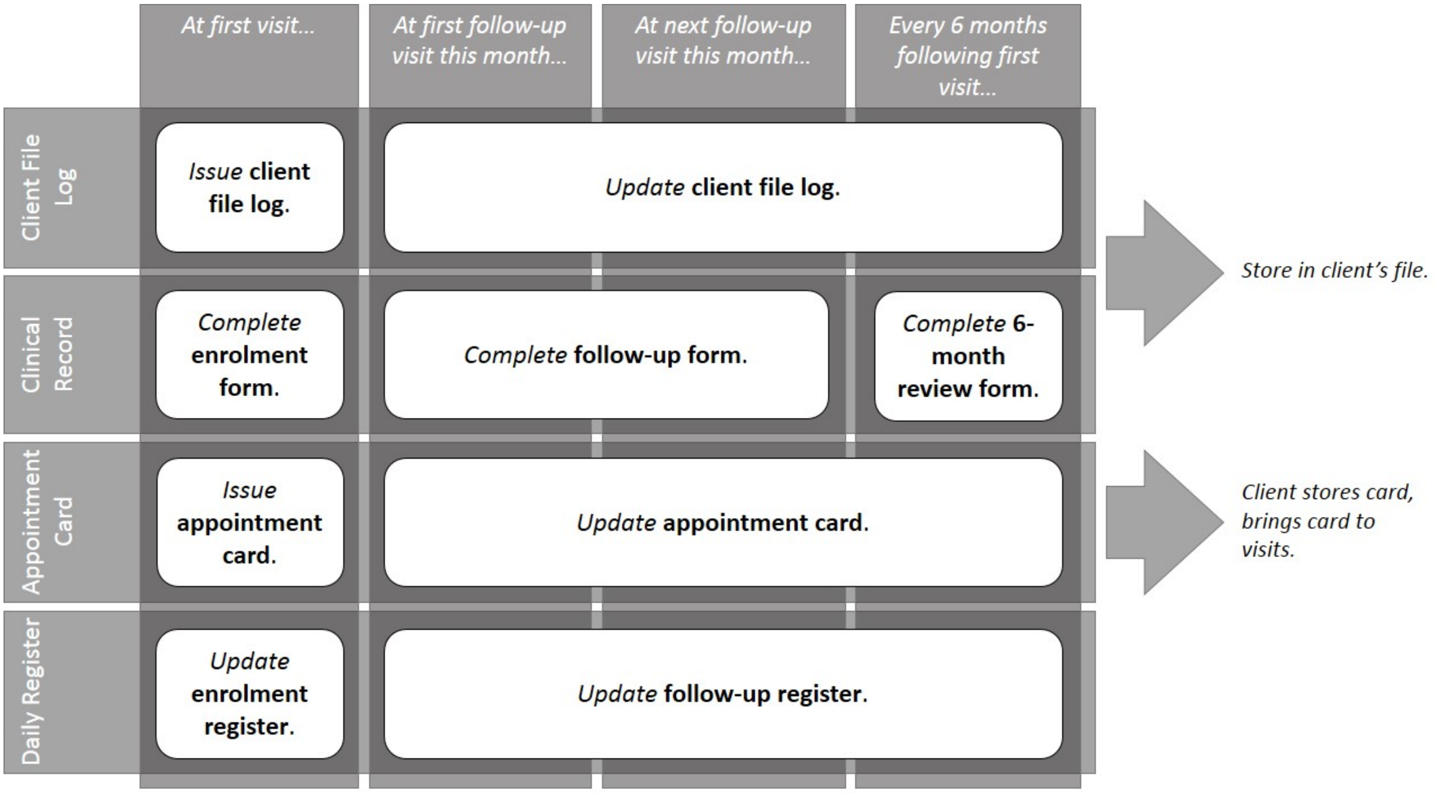

Figure 1. How clinical record keeping responsibilities change between first visit and follow-up visits

D. Filling prescriptions

1. Refer to the most recent form completed for the client in the client's patient file, in order to see the prescription, dosage, and total cost.
2. Fill the prescription with the following information:
   a. Patient ID no.
   b. Payer's name
   c. Today's date

E. Referring clients

1. Whether this is an enrollment, follow-up, or 6-month review visit, you may wish to refer a patient to additional services. Each of the relevant forms includes a section in which you should record the referral.
2. The referral must also be recorded in the "Referrals" section of the **daily register** as an "outgoing referral".
3. Complete the top-half of a **referral form**, and give the full form (top and bottom halves intact) to the patient to take to the receiving facility. Instruct the patient to give the whole referral form to the receiving facility, and to bring the bottom-half only back to your clinic. The top-half remains with the receiving facility.
4. Make a new entry on the "Register of Referrals OUT" table of the **referral register**, recording this referral. When the client does eventually return with the bottom-half (the "back-referral" section), write the date of receipt in the "Date back referral received" column of the register.
5. File the returned "back-referral" section of the referral form in the patient file, using the patient ID no. to locate the appropriate file.

F. Receiving referrals

1. When a new client comes to the clinic with a **referral form**, first proceed with the general process for "Enrolling a new client" (Section IV.A).
2. Ensure that the referral was recorded in the "Referrals" section of the **daily register** as an "incoming referral".
3. Make a new entry on the "Register of Referrals IN" table of the **referral register**, recording this referral.
4. After the client has been enrolled, complete the bottom-half ("back referral" section) of the referral form, and detach it from the top section. Return the bottom half to the patient to bring back to the initiating facility. Store the top-half in the new patient file.

Common Questions

Q1: *What if I find I can't get all of this record-keeping work done each day?*

A1: It isn't worth "cutting corners" with record-keeping in order to save time. If you find you must work late hours in order to serve all your clients and update their records daily, speak with your supervisor about getting assistance. The items marked with an asterisk* can be "task-shifted" to a less-specialized health worker, as long as he or she is well-trained and supervised. Your supervisor may also observe a day in the clinic with you and make suggestions about strategies to save time and improve efficiency.

Q2: *What do I do if an existing client shows up without a card?*

A2: If the client shows up without the appointment card, do not assign him or her a new patient ID no. or create a new file. You can still locate the patient ID no. to retrieve the existing file. Ask the client approximately when he or she last attended the clinic, and scan your daily register from around that time period to search for the client's name.* Each entry in the register should have the date, client's name, and patient ID no. listed, so you should then be able to retrieve the correct patient ID no. and corresponding file. Issue a new appointment card, if necessary, and urge the client to bring it to the next appointment.

Q3: *Who fills out the Data Entry Date on the file log?*

A3: The clinical officer or another supervisor responsible for data entry will fill this section after entering patient data electronically from the files in the form.

Q4: *What do I do if I'm not sure how to record something?*

A4: Never skip a question on any form unless the form specifically advises you to (e.g. on a question that says, "Only complete for epilepsy patients" when you are recording information for a patient without epilepsy). These SOPs and the supporting materials provided should be able to help guide you, but if you're still uncertain, or if you feel that the options provided don't adequately reflect the information you wish to record (e.g. if you've prescribed a drug that isn't listed on the list of drug options available to tick), please consult your supervisor as soon as possible. You may have identified an issue with the tools provided that we need to address in order to ensure that they are as clear and as useful as possible to CPNs.

V. Reporting your services

A. Routine Reporting: HIS data

1. Each month, tally all of the information recorded in the previous month in the **new client register** of the **daily register**, by counting the total number of ticks in each column. Use the **monthly register tally sheet** to help you. Then use this information to fill in the appropriate sections in the **monthly reporting form**, including:
    a. Number of new clients served, disaggregated by age and sex
    b. Number of new clients served, disaggregated by CGI-S score
    c. Number of new clients served, disaggregated by diagnosis
2. Each month, tally all of the information recorded from each *unique* follow-up client in the **existing client register**, by using the **monthly register tally sheet** to separate the information collected from clients' first follow-up visit of the month from information that may have been collected on subsequent visits. Then use the information tallied up from first visits only to fill in the appropriate sections in the **monthly reporting form**, including:
    a. Number of follow-up clients served, disaggregated by age and sex
    b. Number of follow-up clients served, disaggregated by CGI-S score
    c. Number of follow-up clients served, disaggregated by diagnosis
3. Each month, tally up the total number of incoming and outgoing referrals from both the **new client register** and the **existing client register**. Add these together to fill in the last two sections of the **monthly reporting form**:
    a. Number of clients referred into the facility
    b. Number of clients referred out of the facility

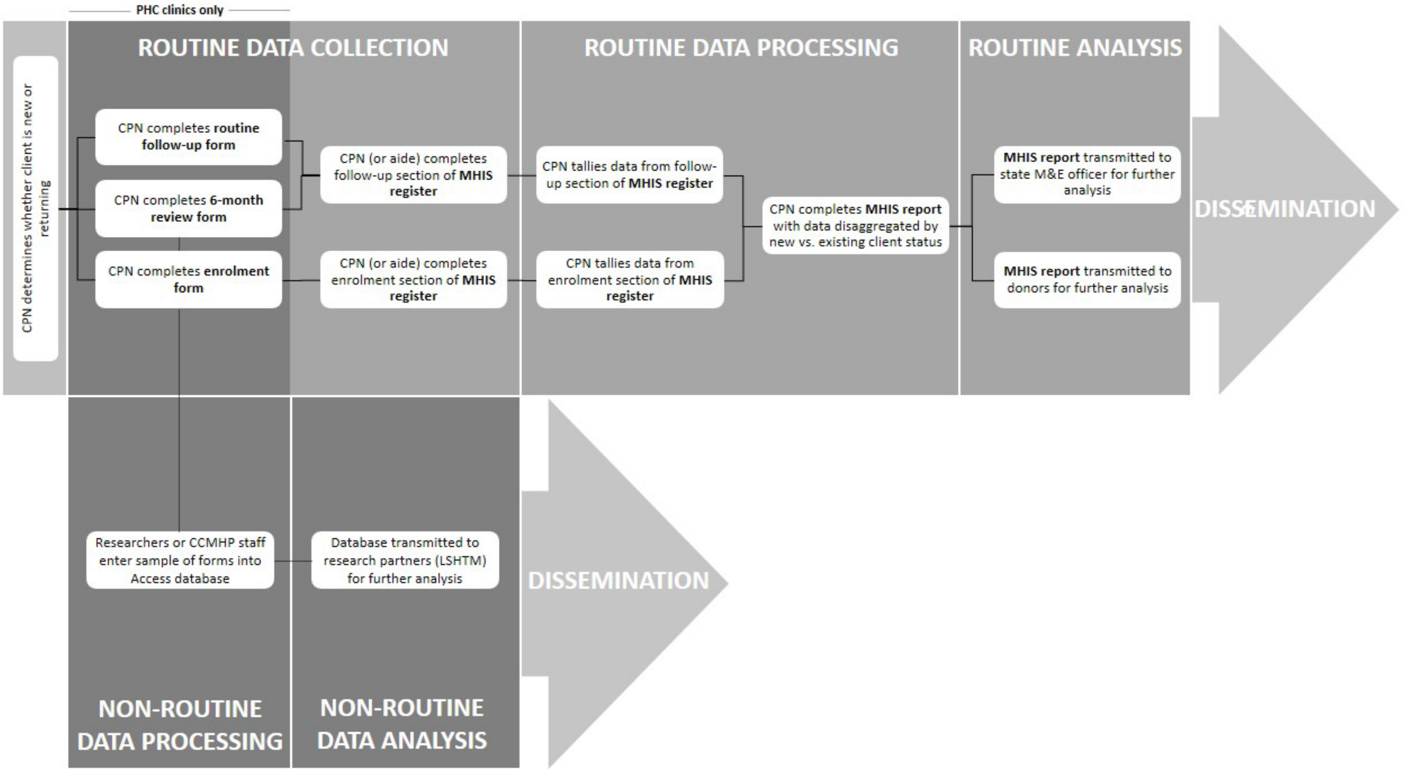

Figure 2. How HIS reporting fits within the M&E strategy

Common Questions

Q1: *Why do I need to report HIS data each month?*

A1: In order to ensure timely reporting of key indicators through State channels, HIS data is aggregated on a monthly basis by the CPN and submitted to both the health center management and to the project. Aggregating the data not only ensures transparency, it also enables you to be responsive to important trends and changes to improve the quality of your services.

For example, a CPN who finds she has received few referrals may decide she needs to visit other health centers in the area to make them aware of her services. Another CPN who notices he receives mostly clients with psychosis will want to request a larger supply of anti-psychotics, and further conduct community outreach to increase detection of clients with other diagnoses like depression or epilepsy.

Q2: *What happens to all the other client data I've recorded in my forms?*

A2: The forms you include in each patient file include a lot of rich data that can help you to remember key information about each client, make clinical decisions appropriate to his or her needs, and judge his or her progress. This information also enables your supervisor to help advice you on a case-by-case basis.

Eventually, this information will also be aggregated and analyzed in order to evaluate the program as a whole. As you can see in Figure 2, shown above, monthly HIS reporting is the routine process we rely on to monitor services routinely. Less routinely, partners will use the rich data you've collected to evaluate the impact this service has on patient outcomes and help build the evidence base for community mental health in Nigeria.

Q3: *What do I do if I think a mistake has been made?*

A3: Be open with your supervisor about any errors you think you may have made in recording or reporting data. The feedback you provide will help to inform further refinement.

Q4. *What will my supervisor want to see?*

A4. One of the roles of your supervisor is to support you both in the actual provision of services and also in how you record and report your services. During supervision visits, he or she will want to see the daily register, monthly report, and a selection of patient files—especially any about which you have questions you would like to ask.

APPOINTMENT CARD

Please present this card to the clinic staff.

**Clinic Name:**						
**Patient Name:**						
**Patient No:**						
**Notes:**						

APPOINTMENT CARD

Please present this card to the clinic staff.

**Clinic Name:**						
**Patient Name:**						
**Patient No:**						
**Notes:**						

APPOINTMENT CARD

Please present this card to the clinic staff.

**Clinic Name:**						
**Patient Name:**						
**Patient No:**						
**Notes:**						

APPOINTMENT CARD

Please present this card to the clinic staff.

**Clinic Name:**						
**Patient Name:**						
**Patient No:**						
**Notes:**						

Appointments

	Date		Date
1		19	
2		20	
3		21	
4		22	
5		23	
6		24	
7		25	
8		26	
9		27	
10		28	
11		29	
12		30	
13		31	
14		32	
15		33	
16		34	
17		35	
18		36	

Appointments

	Date		Date
1		19	
2		20	
3		21	
4		22	
5		23	
6		24	
7		25	
8		26	
9		27	
10		28	
11		29	
12		30	
13		31	
14		32	
15		33	
16		34	
17		35	
18		36	

Appointments

	Date		Date
1		19	
2		20	
3		21	
4		22	
5		23	
6		24	
7		25	
8		26	
9		27	
10		28	
11		29	
12		30	
13		31	
14		32	
15		33	
16		34	
17		35	
18		36	

Appointments

	Date		Date
1		19	
2		20	
3		21	
4		22	
5		23	
6		24	
7		25	
8		26	
9		27	
10		28	
11		29	
12		30	
13		31	
14		32	
15		33	
16		34	
17		35	
18		36	

Patient No. | | | | | | | |

COMMUNITY MENTAL HEALTH CLINIC

Patient File

Clinic: ____

#	Visit Date	Visit Type<br>(Tick one)	Data Entry Date<br>(For clinical officer use only)
**1**		**-Enrollment-**	
2		☐ Follow-Up ☐ 6-Month Review	
3		☐ Follow-Up ☐ 6-Month Review	
4		☐ Follow-Up ☐ 6-Month Review	
5		☐ Follow-Up ☐ 6-Month Review	
6		☐ Follow-Up ☐ 6-Month Review	
7		☐ Follow-Up ☐ 6-Month Review	
8		☐ Follow-Up ☐ 6-Month Review	
9		☐ Follow-Up ☐ 6-Month Review	
10		☐ Follow-Up ☐ 6-Month Review	
11		☐ Follow-Up ☐ 6-Month Review	
12		☐ Follow-Up ☐ 6-Month Review	
13		☐ Follow-Up ☐ 6-Month Review	
14		☐ Follow-Up ☐ 6-Month Review	
15		☐ Follow-Up ☐ 6-Month Review	
16		☐ Follow-Up ☐ 6-Month Review	
17		☐ Follow-Up ☐ 6-Month Review	
18		☐ Follow-Up ☐ 6-Month Review	
19		☐ Follow-Up ☐ 6-Month Review	
20		☐ Follow-Up ☐ 6-Month Review	
21		☐ Follow-Up ☐ 6-Month Review	
22		☐ Follow-Up ☐ 6-Month Review	
23		☐ Follow-Up ☐ 6-Month Review	
24		☐ Follow-Up ☐ 6-Month Review	
25		☐ Follow-Up ☐ 6-Month Review	
26		☐ Follow-Up ☐ 6-Month Review	
27		☐ Follow-Up ☐ 6-Month Review	
28		☐ Follow-Up ☐ 6-Month Review	
29		☐ Follow-Up ☐ 6-Month Review	
30		☐ Follow-Up ☐ 6-Month Review	
31		☐ Follow-Up ☐ 6-Month Review	

MONTHLY REPORTING FORM

Client demographics	Males							Females						
	(0-5)	(6-12)	(13-17)	(18-25)	(26-35)	(36-50)	(51+)	(0-5)	(6-12)	(13-17)	(18-25)	(26-35)	(36-50)	(51+)
No. new client contacts, disaggregated by age and sex														
No. follow-up client contacts, disaggregated by age and sex														

Client diagnosis	No. clients served based on Depression/ Anxiety	No. clients served based on Epilepsy	No. clients served based on Psychosis/ Manic Depression/ Bipolar Disorder	No. clients served based on Alcohol/ Substance Abuse	No. clients served based on Developmental delay/disorders	No. clients served based on Child Mental Disorder	No. clients served based on Dementia
No. new clients contacts, disaggregated by diagnosis							
No. follow-up client contacts, disaggregated by diagnosis							

Client referrals	
Number of clients referred into the facility	
Number of clients referred out of the facility	

Adapted from CBM Interntional Community Mental Health Programme
