# Supplementary material for: Integrating mental health into primary care in Nigeria: Implementation outcomes and clinical impact of the HAPPINESS intervention
Source: Glob Ment Health (Camb). 2024 Jan 12;11:e9. doi: 10.1017/gmh.2024.4 (PMC10882178; doi:10.1017/gmh.2024.4)
Supplement: Iheanacho et al. supplementary material 2 — Iheanacho et al. supplementary material [file S2054425124000049sup002.pdf]

The HAPPINESS Project Drug Revolving Fund (DRF)

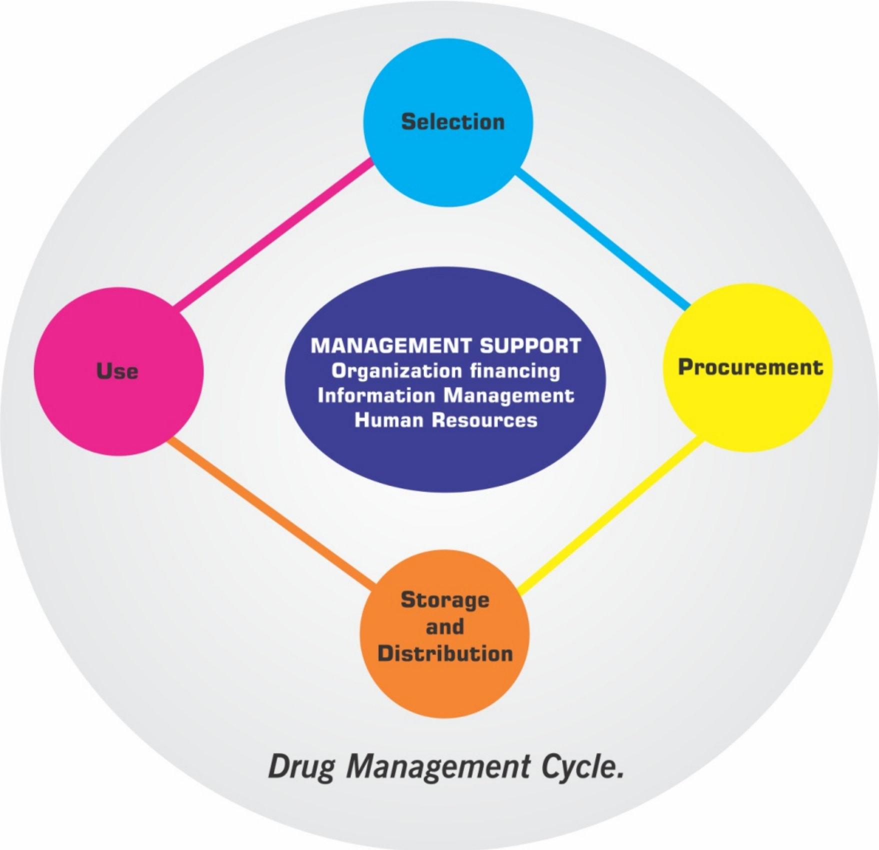

Management:

The HAPPINESS Project DRF will be managed by the HAPPINESS Healthcare Foundation within existing platform of Imo State Primary Care Development agency. The board of trustees will seek the services of a pharmacist who will oversee the day-to-day administration of the DRF with the HAPPINESS Project coordinator.

Financing:

Funding for the DRF will be included as a line item in the HAPPINESS Project 2019 and subsequent years budget.

Direct medication donation from donor agencies will be rolled into the DRF and priced at same rate as purchased medications to maintain the supply chain.

Accounting records:

At the local site and central administration level, the DRF manual on financial management and accounting system that tracks supply, utilization, stocking, re-stocking and finances will be used. Accounting records will be properly and promptly updated.

Internal and external audit:

External audit will be done at the central supply level while at the facility level an internal audit will be done with reporting directly to the head of the health center.

Incentive payment:

We anticipate that the DRF operations will increase the overall workload of the clinics. The clinicians at the primary care clinic will be paid a form of incentive to ensure the success of the scheme.

Cash and carry:

All sales of drugs should be on cash and carry basis. No credit sales. Requisition system will be the "Pull" system to avoid the expiring of drugs and other health commodities. Cash realized from the sale of drugs will be paid into the project account. Both the health worker and project coordinator will acknowledge in writing payment made for drugs by the health worker. The health workers will remit payments for drugs dispensed at monthly intervals. For easy reference, documents should be numbered and put into different files.

Training:

Opportunities for the training of operators of the DRF will be provided regularly to enhance the quantity and quality of services rendered.

Management Information System (MIS):

The appropriate indicator forms developed for the purpose of monitoring the DRF will be used as required. The information derived from the exercise will be appropriately disseminated and used to improve the system.

Pilferage/Theft:

Acceptable loss due to pilferage will not exceed 2% while loss due to breakages and stock expiry will not exceed 3%.

Pricing:

Retail pricing at the facility level will consider purchase price, transportation charges and markup to cover administrative and other costs.

Steps and Procedures in Drug Revolving Fund

1. Selection of Drugs in HAPPINESS Project DRF Scheme will be based on the following factors:

- Keeping costs of drugs and dosage forms affordable and cost-effective so as to optimize the use of financial resources.
- Having drugs available for the treatment of the most prevalent MNS disorders at the primary care levels
- Consistent availability of safe, effective and good quality drugs.

Drugs will be selected based on the Nigeria Essential Drug Lists (EDLs) which used the WHO model as a template. The EDLs is based on National Health Policies and, on National Drug Policies.

The International Nonproprietary Names (INNs), also known as generic names, will be used in identifying selected drugs. However, the choice of drugs by generic names requires the existence of an effective Drug Regulatory Authority (NAFDAC) to ensure the availability of good-quality, safe, effective and affordable drugs. The HAPPINESS Project DRF will reflect the needs of the Primary Health Centers (PHCs), which is the main focus of this project. In general, PHCs usually have ten to thirty drugs from the national essential drugs lists. This small number of drugs makes procurement, storage and distribution easy.

2. **Procurement of Drug Requirements**

Our procurement process will be based on drug quantification and tender management. We will start with selected drugs, dosage forms and available financial resources. The following procedures will be adopted in procuring drugs:

- Estimating quantity of each drug product required for a given period,
- Finding out the prices of the different drug dosage forms required
- Allocating funds for each drug dosage form depending on:
- Priority nature of drug and dosage form,
- Available finances

3. Estimation of Drug Requirements

The estimate of the drug and dosage forms required for a given period will be undertaken to avoid shortages (out of stock), ensure credible health care service, prevent excess stock and avoid waste (loss or mismanagement of financial resources). The following factors influences choice and quantity of drugs:

- Population which the health center serves;
- MNS Disorders pattern;

- Monthly (rate of) drug consumption;
- Knowledge of quantity of each dosage form that is regularly consumed
- Delivery (lead) time;
- Time lag between placing orders and receiving the orders;
- Request indicator (re-order level);
- Quantity of drug product that serves as a signal for re-ordering.

The maximum quantity of drugs held in stock will be determined by distance and accessibility from the central office, size of the health center store and number of clients (patients) visiting the health center. The following three factors will be considered as the basis for calculating the appropriate quantity of a particular drug to be ordered.

-Delivery (Lead) time

It is the estimated time between ordering drugs and receiving the drugs. It is also the time when new stock is ordered and when it is received and available for use.

Delivery time may be days or weeks; it may be affected by the following:

- Poor road conditions, particularly in the rainy season
- Transportation difficulties
- Consumption rate of drugs

Receipt of drugs at the health center

In receipt of drug at the health center, the following should be noted:

1. Check that the quantity issued actually corresponds to the quantity indicated on the stores requisition/delivery (issue) form.
2. Check off each drug after checking.
3. Take note of the unit price of each drug and compare it to the previous unit price.

4. Check that all original boxes, tins or bottles are unopened and are in good condition.
5. Check the labels and ensure that there are no expired drugs being received.
6. Any drugs already expired or soon to expire that cannot be consumed before expiration or drugs not in good condition should be returned for destruction or redistribution to other Center.

Requisition/Delivery (Issue) Form

This form is to be filled when the PHC dispensary is ordering items from the central office.

In filling the forms, one must ensure that the following items are filled in correctly.

1. Name of drug and dosage form;
2. Unit of issue and quantity requested;
3. The requisition number (it is preferable to begin with a new number each year, e.g.)
4. The name of the dispensary and the date the requisition was made;
5. The name and signature of the health worker making the requisition
6. Name and signature of the health worker making the requisition

APPENDIX

Drug Revolving Fund HAPPINESS Project

Cost of Medications (For HAPPINNESS office only)

Medication	Unit cost	HAPPINESS Project price	Dispensing Price PHC	Total Profit margin/tab	Margin per Tablet PHC
**Drugs procured**					

Drug Revolving Fund HAPPINESS Project

Medication supplies office use only

Medication	Quantity supplied	HAPPINESS Project price	Dispensing Price PHC
Drugs procured on			

Drug Revolving Fund HAPPINESS Project

Medication supplies HAPPINESS Project Office **Centre:**

Medication	Expiry date	Quantity supplied	Date supplied	Value of total expected cost	Signature of supplying officer	Signature of receiving officer
**DATE**						
Tabs Amitriptylline						
Tabs Carbamazepine						
Tabs Chlorpromazine						
Tabs Benzhexol						
Tabs Haloperidol						
**Total Expected Cost**						
Total amount received 1						
Total amount received 2						
Total amount received 3						
Total amount received 4						
Total amount received 5						
Total amount received 6						
Total amount received 7						

Drug Revolving Fund HAPPINESS Project

Medication supplies HAPPINESS Project Office **Centre:**

**Medication**	**Expiry date**	**Quantity supplied**	**Date supplied**	**Value of total expected cost**	**Value due HAPPINESS PROJECT**	**Value due Health Worker**
**DATE**						
Tabs Amitriptylline						
Tabs Carbamazepine						
Tabs Chlorpromazine						
Tabs Benzhexol						
Tabs Haloperidol						
**Total Expected Cost**						
Total amount received 1						
Total amount received 2						
Total amount received 3						
Total amount received 4						
Total amount received 5						
Total amount received 6						
Total amount received 7						

Drug Revolving Fund

Medication cost (For PHC notice board)

Medication	Dispensing Price
Tabs Amitriptylline	10.00 Naira each
Tabs Carbamazepine	15.00 Naira each
Tabs Chlorpromazine	12.00 Naira each
Tabs Benzhexol	10.00 Naira each
Tabs Haloperidol	10.00Naira each

<u>HAPPINESS PROJECT DRF INVOICE</u>

NAME OF CENTRE:

NAME OF STAFF:

S/N	DATE	NAME OF PATIENT	DRUG DISPENSED	QUANTITY	TOTAL COST

HAPPINESS PROJECT DRF INVOICE **Health Centre**

For health centre staff

S/N	DRUG	QTY SUPPLIED	VALUE	NAME OF RECEIVER /CENTRE	DATE	NAME AND SIGNATURE OF SUPPLYING OFFICER

BIN CARD

HEALTH CENTRE---
ITEM--
DOSAGE---
QUANTITY OF TABLETS /UNIT-------------------------------------

Date	Ref. No.	From/To	Quantity In	Quantity Out	Balance	Unit Price	Remar k	Signature
